# cmFSM: a scalable CPU-MIC coordinated drug-finding tool by frequent subgraph mining

**DOI:** 10.1186/s12859-018-2071-z

**Published:** 2018-05-08

**Authors:** Shunyun Yang, Runxin Guo, Rui Liu, Xiangke Liao, Quan Zou, Benyun Shi, Shaoliang Peng

**Affiliations:** 1 College of Computer Science and Electronic Engineering & National Supercomputer Centre in Changsha, Hunan University, Changsha, 410082 China; 2 School of Computer Science, National University of Defense Technology, Changsha, 410073 China; 3 Department of Oncology, The Second Xiangya Hospital of Central South University, Changsha, 410011 China; 4 School of Computer Science and Technology, Tianjin University, Tianjin, 300072 China; 5 School of Cyberspace, Hangzhou Dianzi University, Hangzhou, 310018 China

**Keywords:** Frequent subgraph mining, Bioinformatics, Memory constraints, Isomorphism, Many integrated Core (MIC)

## Abstract

**Background:**

Frequent subgraphs mining is a significant problem in many practical domains. The solution of this kind of problem can particularly used in some large-scale drug molecular or biological libraries to help us find drugs or core biological structures rapidly and predict toxicity of some unknown compounds. The main challenge is its efficiency, as (i) it is computationally intensive to test for graph isomorphisms, and (ii) the graph collection to be mined and mining results can be very large. Existing solutions often require days to derive mining results from biological networks even with relative low support threshold. Also, the whole mining results always cannot be stored in single node memory.

**Results:**

In this paper, we implement a parallel acceleration tool for classical frequent subgraph mining algorithm called cmFSM. The core idea is to employ parallel techniques to parallelize extension tasks, so as to reduce computation time. On the other hand, we employ multi-node strategy to solve the problem of memory constraints. The parallel optimization of cmFSM is carried out on three different levels, including the fine-grained OpenMP parallelization on single node, multi-node multi-process parallel acceleration and CPU-MIC collaborated parallel optimization.

**Conclusions:**

Evaluation results show that cmFSM clearly outperforms the existing state-of-the-art miners even if we only hold a few parallel computing resources. It means that cmFSM provides a practical solution to frequent subgraph mining problem with huge number of mining results. Specifically, our solution is up to one order of magnitude faster than the best CPU-based approach on single node and presents a promising scalability of massive mining tasks in multi-node scenario. More source code are available at:Source Code: https://github.com/ysycloud/cmFSM.

## Background

### Frequent subgraphs mining

Frequent subgraphs mining in a collection of graph objects is a very difficult challenge, especially in the important application area “Bioinformatics” where it can help finding new drugs in pharmacological compound databases or core functional structures in biological networks. Subgraph mining problem is difficult to solve because arbitrary graph structures must be generated and matched. As isomorphism testing is a hard problem [1], subgraph miners are exponential in memory consumption and execution time.

Lin [2] have summarized that the problem of frequent subgraph mining mainly consists of two categories: (i) frequent subgraph patterns ought to be found in different regions of one large graph of massive scale; (ii) frequent subgraph patterns should be found within a large-scale collection of middle-sized graphs. The first case is usually adapted to social network domain, and the second case is usually adapted to the areas of computational pharmacology and bioinformatics. Both categories share several common challenges. For example, large data input size with relative low support threshold can lead to huge number of mining results, which may exceed the memory of a single machine, and require vast amounts of runtime. Given these characteristics, parallel techniques are presented as a promising solution to solve these challenges.

The objective of this problem is to find subgraphs that occur with support higher than a threshold *θ*, i.e., 0 ≪ *θ* ≪ 1. Several solutions have been put up with for the first case in either serial CPU-based techniques [3–5] or parallel computing (MapReduce, MPI, Spark) framework [6–9] and GPU [10]. However, we mainly focus on the second case, which is more practical in the field of bioinformatics and known as transaction setting [11].

### Related work

In the transaction scenario, miner/frequent subgraph mining algorithm recursively generates all possible refinement extensions from empty graph by adding edges and nodes to already generated refinements. Then, isomorphism test will be performed of each new possible refinement to determine if it appears frequently. Early miner/frequent subgraph mining algorithms generated refinements in a Breadth First Search (BFS) way, e.g., AGM [12], and FSG [13]. However, the Depth First Search (DFS) approaches need less memory and almost show better performance. [14] have summarized three main subproblems (i.e. Purposive refinement Efficient enumeration and Focused isomorphism testing) have to be solved of efficient miners and made a quantitative and detailed comparison of some typical dfs-algorithms, e.g., MoFa [15], FFSM [16], gSpan [17] and Gaston [18], and some special extensions of them, e.g., CloseGraph [19], showing them attacking the three subproblems quite differently. Thus, the dfs-algorithms will be mainly used for comparative analysis with our work. A follow-up work [20] is more focus on accelerating the mining process.

All solutions discussed above are single-core serial version. When they come to large-scale mining problems, they may be difficult to meet time requirement. To solve this problem, SUBDUE [21] develops a shared-memory parallel approach by using the minimum description length (MDL) principle and embodies in the SUBDUE system. Further, [22] proposes parallel mining strategy in a multi-core system and partitions the mining tasks among multiple shared-memory processors. To some extent, these studies make full use of the machine resources on single node to accelerate the mining process.

Another problem is all these approaches are memory-based, and assume that the collection, intermediate data and mining results fit in memory. However, as data size increases and especially support threshold decreases (the scale of mining results grows exponentially), the assumption no longer reach. To address this limitation, some disk-based solutions have been proposed, e. g. ADI-Mine [23]. However, these approaches face significant overhead of accessing the data. The same as a disk-based solution, PGM [24] propose a data partition approach.

The work in IFSM [11] is relatively early to employ MapReduce [25] framework for mining frequent subgraphs. Specifically, it first determines local support for all possible single-edge subgraphs by mapping a part of the graph collection to each worker. Secondly, a reduction phase will determine the global support for each subgraph, and discard those candidates that do not reach the global support threshold. The solution continues to deal with the double-edge subgraphs, triple-edge subgraphs and so on. Similar with IFSM [11], FSM-H [26] and mrFSM [27] are also developed on MapReduce framework by an iterative method. Relatively, [27] pay more attention to the load balancing in each iteration. However, because MapReduce is not suitable for iterative computing, which may result in a lot of IO and serialization overhead, these approaches on MapReduce still create significant performance problem.

The more outstanding work so far on MapReduce framework is MRFSM [28]. It does not adopt iterative method, but the whole process is divided into two MapReduce stages: filter and refinement. The filter stage prunes based on the probability of support and outputs local frequent subgraphs in the local dataset which is divided to each machine. The refinement phase intelligently translates the local support which is gotten from the filter phase into a global support to integrate the final results. Because there are no iterations, it presents a better performance than IFSM [11], FSM-H [26] and mrFSM [27]. However, the implementation code of MRFSM is not purely native java program, most program is coding by C++, and then use Hadoop Streaming to adapt to MapReduce framework to complete distributed mining. As a result, the performance of the MRFSM will be severely restricted because of limited data exchange capacity using standard I/O and redundant data type conversions. Moreover, when the support threshold is low enough, the massive mining results tend to far exceed the single-machine memory. In this respect, because the refinement stage distributes all candidate subgraphs to all machines, this stage can easily cause severe memory pressure on each machine when a large number of candidate subgraphs cannot be filtered due to the low support threshold. Thus, MRFSM [28] may not be able to cope with scenarios with massive mining results due to low support threshold.

In contrast, our tool is implemented by native C++ program with several efficient parallel techniques, e.g., MPI and OpenMP, to maximize performance. Also, every node will not hold all candidate subgraphs but carry out its own mining process to get local final results so as to easily handle the scenarios with massive mining results.

## Methods

cmFSM realizes parallelism of multiple levels and multiple granularities and utilizes MIC as accelerator. Multi-threading is implemented using OpenMP aimed at hotspots of mining process. Four kinds of static task dividing strategy and a supervisor-based dynamic task dividing strategy are implemented by MPI to achieve best load balancing. Further, we used MICs in offload mode only to transfer double-edge frequent subgraphs and back up complex data structures redundantly to avoid the bottlenecks caused by excessive transmission. By making full use of the multi-core computing capacity of MIC, we can achieve a desired effect of execution speed in the scenario of CPU and MIC collaboration.

### OpenMP parallelization on single node

1. The strategy of parallelization

The general dfs-algorithm of frequent subgraph mining uses a recursive approach to deal with the hotspot of mining process, which is very difficult to control the parallel granularity. Also, a simple function call can continuously find out a large number of mining results because we cannot pick out or predict the depth of recursive process. This is bound to lead to load unbalance among different mining tasks.

In order to solve this problem to achieve better effect of parallelization by OpenMP, we adopt a fine-grained parallel strategy. Specifically, we translate the common recursive mining process into a BFS loop mining process by one-edge growth of several layers, so as to implement the parallelization on the granularity of one-edge growth. The operation consists of two specific parts: (i) judgment of minimum DFS code and (ii) right-most extension. Overall, the computing scale of this operation is not too small, so that there is no possibility that most CPU resources is used in thread scheduling because the parallel granularity is not big enough. At the same time, the tasks of the two parts are specific and similar so as to easily achieve a good load balancing by dynamic scheduling strategy of OpenMP. Moreover, there is no need of system to help us manage the stack because recursive processes are replaced by loops, which may lead to additional acceleration.

Take gSpan [15] as an example, the following pseudocode compares original Algorithm 1 with new parallel Algorithm 2.

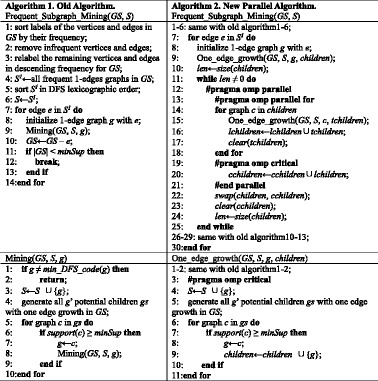



As the pseudocode shown above, in order to complete the parallelization, we apply for four new categories of buffer: children, tchildren, lchildren, cchildren. The children are used to record the set of subgraphs obtained from each level extension where the subgraphs in same level have same number of edges. When children is not empty, the next level mining process will be carried out. It is a global variable and will be used sequentially. The tchildren is a local variable within single-thread tasks, recording the subgraphs obtained from one-edge growth of each subgraph. The lchildren is also a local variable within single-thread tasks, but it is a summary of the results of all one-edge growths for each thread and gotten from the union of tchildren in every iteration. The cchildren also records the set of subgraphs obtained from each level extension. At the end of single-thread tasks, the lchildren will be summarized to cchildren in the critical area. Also, the cchildren and children will be exchanged out of the parallel area in order to carry out the next level iterative mining. It is worth noting that the meaning of the existence of cchildren is that we cannot directly summarize lchildren to children, because at the parallel computing scenario we cannot make sure all extension tasks in every thread are over at the same time. The thread not yet complete tasks will continue to use the data of children, which may lead to failures.

2) 2) Memory Management Deep Optimization

**Fig. 1:**
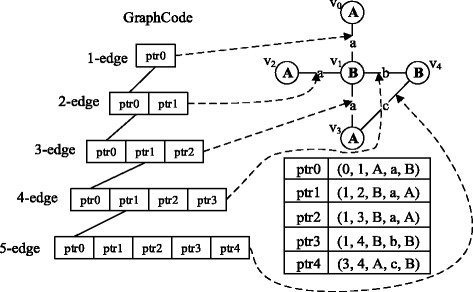
Memory Reuse. The left side shows the frequent subgraph extension process in the form of graph code. The upper right subfigure is the original subgraph. Below it lists the codes of each edge and their corresponding pointers

The main challenge of frequent subgraph mining is the memory constraint. In order to achieve the purpose of memory reuse and the efficient utilization of memory space, we adopt the strategy named “apply dynamically & store pointers”. Specifically, when the subgraph is extended, the program applies edges dynamically and stores edge pointers rather than actual edge objects in the graph code structure, so that the new frequent graphs share most edges with their ancestors, which will lead to a significant saving in memory space. The schematic diagram is shown in Fig. 1. It can be easily seen that only the edge pointers are stored in the graph code and each edge has only one instance in memory, so as to achieve the purpose of memory reuse.

### Multi-node multi-process parallel acceleration

1. The strategies of task division

The biggest challenge of multi-node program is the communication overhead. To solve this problem, a coarse-grained parallel strategy is used among processes through dividing single-edge frequent subgraphs by MPI. The main tasks of each process are completely independent to avoid a large amount of communication overhead, and then each processes will write out its corresponding results in its own node to avoid the single-node memory pressure causing by massive mining results. We should notice that all output files can be easily merged to form the whole results. Further, combining with the multi-thread work on single node, our implementation allows to generate a second level of parallelization by creating multiple threads per MPI process to achieve a good performance.

However, this coarse-grained strategy is not conducive to load balancing, which is easily lead to data tilt and cannot make full use of system resources to achieve the best performance. Therefore, we design and provide four kinds of static task dividing (i.e. equality, single, increment, circle) strategies and a supervisor-based dynamic task dividing strategy based on different characteristics of datasets to achieve load balancing as much as possible.

- static division___equality

A simple strategy is to divide the single-edge frequent subgraphs equally. However, we found that the load is extremely unbalanced after the experiment, and it is easy to achieve bottlenecks. Also, a lot of mining tasks are concentrated on the front of the nodes, and they will also pick out most results. This is because these single-edge frequent subgraphs will be sorted in descending order in the pretreatment stage. The more front they sorted, the more frequent they are and also the more frequent subgraphs they may pick out. And those sorted behind will be closer to the support threshold, they may no longer a frequent subgraph after one-edge growth even one time, so that they may pick out a little results and stop tasks early. Moreover, we find that the scale of results is decreased exponentially as the pretreatment order. Therefore, this intuitive strategy in most cases are difficult to achieve load balancing.

b) static division___increment

In order to solve the problem of equal division, we propose an incremental task allocation strategy. Specifically, the first node gets only one graph, the second node gets two graphs, the third node gets three graphs, and so on, and the last node gets all remaining graphs. Implementation of this strategy actually improves the performance and achieve a better load balancing.

c) static division___single

Actually, when the dataset is big enough, although the tasks of single-edge subgraphs ranking behind will drastically reduce, the gap among the tasks of top-ranked single-edge subgraphs is not obvious. In this case, incremental strategy will lead the relative top nodes to undertake more tasks, so that the load is still not balance. For this scenario, we proposed a single task allocation strategy, in which we assign all preceding nodes only one single-edge frequent subgraph, and the remaining subgraphs are allocated to the last node. This strategy sometimes can achieve a better performance.

d) static division___circle

Considering the single strategy may make a large number of tasks focused on the last node when the dataset is big enough but the degree of parallelism is not particularly high, we proposed a circle task allocation strategy, in which we first allocate all nodes a round of single-edge subgraphs in turn, and then we allocate all nodes a round of graphs in reverse order, and then we allocate all nodes a round of graphs in turn again, and so on until all single-edge frequent subgraphs are allocated to their corresponding nodes. This strategy is expected in most cases to achieve better load balancing.

e) dynamic division___supervisor-based

In fact, because the mining process is very complex and it is difficult for us to predict or measure the scale of tasks, these static task division strategies certainly cannot adapt to all practical scenarios. A more ideal method is to use dynamic division strategy based on task queue, in which we first allocate a round of single-edge subgraphs in turn to all nodes, and then one of the remaining graphs will be allocated to the node that finishes tasks earliest to carry out mining process until the end of all tasks. In theory, this strategy can always achieve a better load balancing compare to static division strategies.

In order to implement this dynamic strategy, we treat process0 as a supervisor, which manages all tasks uniformly. When other processes finish their current tasks, they first ask process0 for a remaining single-edge frequent subgraph. The process0 will search its task queue and reply to them. When the task queue is not empty, the process0 will allocate a new single-edge frequent subgraph to other processes, otherwise, it replies − 1 and counts. When the count reaches the number of process, the process0 will end its work. On the other hand, when other processes receive − 1 once, they will also end their work.

**Fig. 2:**
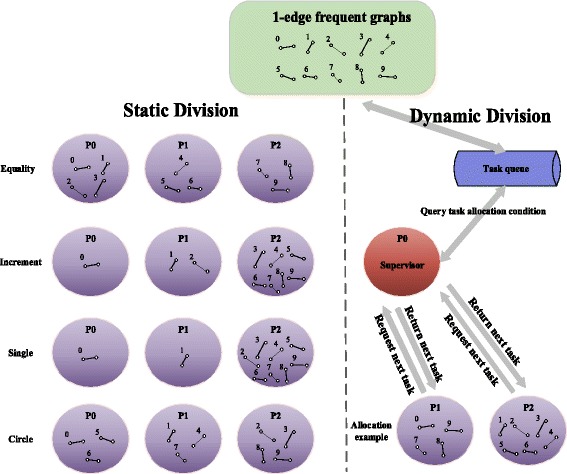
Different kinds of task dividing strategies. Note that the equality division is not absolute, but each process holds the number of single-edge frequent subgraphs will be the same or keep the difference that no more than one. At the same time, the example of dynamic division in this figure shows only a possible result which is not necessary in this case

The Fig. 2 shows an example of five division strategies. The dynamic strategy can always achieve a better load balancing than static strategies, but the overall performance is not necessarily more optimal because of other operations such as request, wait, communication and synchronization. Thus, users can choose all these strategies. However, in most cases it is recommended to adopt dynamic strategy.

2) Remove multi-node redundant results

Another problem in multi-node scenario is the redundant results. From Algorithm 1, it is not difficult to find that the original single-edge frequent subgraph must be deleted after its extension tasks. This is to avoid to consider the high-frequency single-edge subgraphs which have been used in the following mining process, which may lead to redundant results. This possibility can result in some difficulties in multi-node scenario. Because each process can only handle its own single-edge frequent subgraphs in current mining process, the high-frequency single-edge subgraphs in other nodes which should not be considered will not be deleted.

We extend the parallel algorithm to multi-node scenario and show it in the following Algorithm 3.

In order to extend the parallel algorithm, we notice that the single-edge subgraphs which are allocated to every node are also be handled in turn. This characteristic make it possible for us to remove high-frequency single-edge subgraphs which priori to current object before the mining process.

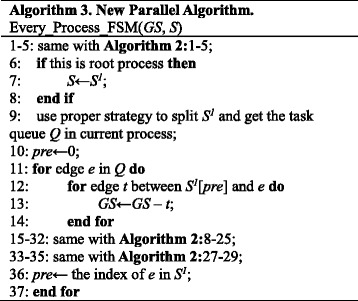



### CPU-MIC collaborated parallel optimization

1. Collaborated Parallelization of cmFSM

The collaboration among CPUs and MICs we employ a medium-grained parallel strategy. In detail, we divide the double-edge frequent subgraphs among CPUs and MICs, which are gotten from one-edge growth of initial single-edge frequent subgraphs in every process. Also, we adopt offload mode to transfer double-edge frequent subgraphs from host to MICs. By tolerating appropriate communication overhead and making full use of the multi-core computing capacity of MIC, we can achieve a desired effect of load balancing and computing speed.

**Fig. 3:**
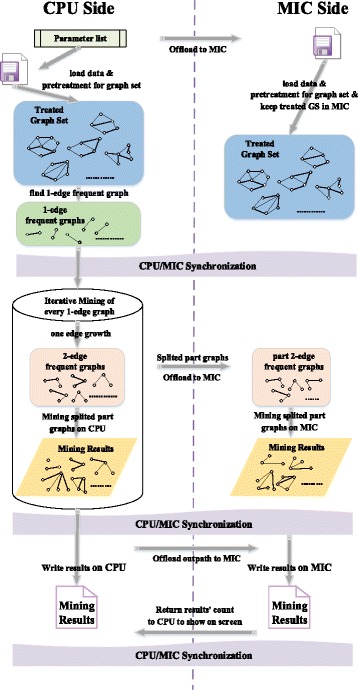
Framework of CPU/MIC collaboration. There are mainly three stages of MIC process: (i) Data loading and pretreatment (ii) Iterative mining process and (iii) Output the results. All these tasks are collaborated with CPU at the same time. Moreover, at the end of each stage, the two devices will synchronize their tasks to ensure the correctness and efficiency of the program

The Fig. 3 shows interactive process between CPU and MIC to achieve the ideal collaboration. It is worth noting that the coarse-grained strategy is not used because it is difficult to effectively reach load balancing under this strategy. Also, the single-edge frequent subgraphs are allocated to each process are not definitely able to be divided reasonably. For example, only one single-edge frequent subgraph may be allocated to some processes in many cases. Coupled with the truth that there is a difference of computing capacity between CPU and MIC, load balancing will be a great challenge. On the other hand, the fine-grained strategy also should not be considered, because it is not shared memory between CPU and MIC. There must be a huge scale of communication overhead to transfer and divide the graphs by offload mode. Thus, this strategy is also not conducive to improve the overall performance.

2) Memory Reuse

The memory of one MIC card is approximately 5 Gigabytes, which cannot be matched to the general node memory. Also, the speed of memory allocation and release is still slower than CPU. Experimental tests show that allocating 1 Gigabyte of memory on MIC takes approximately 5 s. Therefore, you must reduce the frequency of memory allocation and release on MIC and maximize possibility of memory reuse.

In addition to the usage of “apply dynamically & store pointers” strategy we introduced before, cmFSM reduces the memory allocation time on MIC by memory reuse. We create a counter JobCount to record the job number. If count = 1, it uses “alloc_if(1) free_if(0)” to allocate memory for the array and object listed in the offload segment. When count > 1, it employs “alloc_if(0) free_if(0)” to reuse the memory. Until the last time, it adopts “alloc_if(0) free_if(1)” to release the memory after the operation is completed. By this way, we can minimize the frequency of memory allocation and release on MIC.

On the other hand, when the dataset is relatively large and the mining process is deep enough, even if a whole extension process of one single-edge frequent subgraph can use up the memory on MIC. In this scenario, it is not suitable to transfer all the data to be mined once and then mining their results. Therefore, instead of uploading all the double-edge frequent graphs at one time, we adopt an iterative method to upload only a part of the graphs obtained from the same single-edge frequent graph at one time to facilitate data compression and save the memory space on MIC, which can be clearly seen from Fig. 3.

3) Data Transmission Optimization

**Fig. 4:**
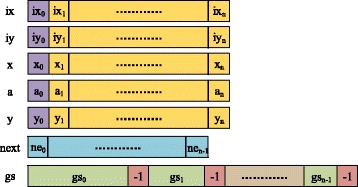
Data structure used in offload mode. The format of n double-edge frequent subgraphs that are supposed to be transfered in an iteration

Although the C++ STL container and class is supported by MIC, the ICC compiler does not support the use of offload mode to transfer these structures. It can only support the basic datatype and array without pointers. Thus, we adopt the strategy named “dismantle & restore” to transfer the objects. The Fig. 4 shows the format of n double-edge frequent subgraphs that are supposed to be transfered in an iteration.

We first dismantle these objects and integrate their elements to make those of same kind are stored in the same successive buffer. Then, we transfer these buffers to MIC by offload mode and allocate memory of original objects on MIC. Finally, we restore these objects on MIC by filling corresponding elements from these buffers. This is no doubt a troublesome process, but fortunately we only need to transfer some double-edge frequent subgraphs with the same first edge in an iteration according to the above idea.

From the Fig. 4, we can find that there are seven buffers are used in transmission process. Because the format of edge code is a quintuple (ix, iy, x, a, y), the first five buffers are used to transfer edges. The elements with subscript 0 represent the first common edge. The following n elements represent the second edges of every subgraph. The next buffer represents the number of nodes of n subgraphs. The gs buffer is the numbered lists of original graphs where this subgraph appeared in dataset. Because the number begins from 0, we simply employ − 1 to separate these lists. From this formation, we can organize and transfer data between CPU and MIC efficiently.

On the other hand, some complex data structures will be used in the whole mining process, such as original graph set after the pretreatment, which may lead to unbearable transmission overhead and memory allocation overhead. Therefore, we back up complex data structures redundantly which are reusable and difficult to transfer to maximize performance. In detail, at the beginning we only transfer the analytical parameters to MIC, the coprocessor can read data and construct graph set by itself based on these parameters, which is not the hotspot of calculation and can be quickly completed on the MIC. Also, in many cases the mining results is too big to transfer by offload mode. Thus, at the end, instead of returning all mining results to CPU, we only return the count of results to show the overall consequence on CPU. Specific mining results will be directly written on MIC. These files can be easily merged to get the whole results. The two stages are clearly illustrated in Fig. 3.

4) Load balancing and data division among CPU/MICs

According to the previous strategy, only the double-edge frequent subgraphs obtained from the one-edge growth of the same single-edge frequent graph will be transferred to MIC in an iteration. If the process continues to mine from this layer, the scale of calculation is supposed to be greatly closer than from single-edge subgraphs. Considering the computing capacities of CPU and MIC are still close in our environment after tests, we simply adopt a static strategy using interval division and make the host device with slightly stronger calculation ability start first, which is because the front double-edge subgraphs theoretically still have more potential to extend, to achieve an efficient load balancing among CPUs and MICs.

**Fig. 5:**
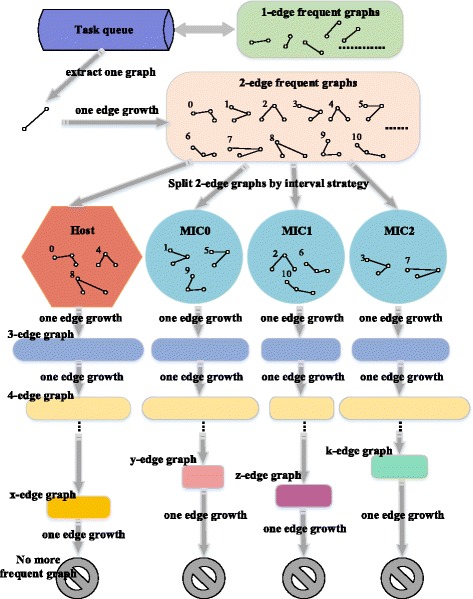
Data division among CPU/MICs and Mining Schematic. The data division and CPU/MIC collaborating mining process in an iteration

The data division and CPU/MIC collaborating mining process in an iteration is shown in the Fig. 5. Taking into account the truth that there are three MICs on single node of Tianhe-2, we take three MICs as an example in Fig. 5. Multiple CPUs in single node share memory, we can manage their computing resources uniformly and call them Host. The method of interval division can be clearly seen from Fig. 5. At the same time, the mining depth or scale of calculation on each device cannot be sure, but all their processes will end until there are no more new frequent subgraphs after one-edge growths. In the multi-node scenario, we just allocate every node different single-edge frequent subgraphs to form different task queues. There are no more other differences.

## Results

We have evaluated the performance of cmFSM under five aspects: (i) parallelization on single node, (ii) multi-node division strategy, (iii) efficiency of multi-node multi-thread acceleration, (iv) CPU/MIC collaboration and (v) multi-node CPU/MIC collaboration.

### Setup and dataset

The cmFSM was implemented in C++ using the STL and compiled with the –O2 compilation flag. The first experiment is performed on a high performance server which consists of 8 Xeon E7–8800 v3 18 core CPU processors with 2 Xeon Phi 3120 57 core coprocessors and 2 K40 M GPU. It has a 2 TB memory in total and the operation system is Red Hat Enterprise Linux Server release 7.1. The next four experiments are performed on the Tianhe-2 supercomputer. The configuration is listed in the following Table 1.

**Table 1:** Tianhe-2 supercomputer Configuration

Hardware	Index
Node number	16,000
Computing node	2 Xeon E5 CPUs and 3 Xeon Phi coprocessors
CPU memory	64 GB
Xeon Phi memory	8 GB
Communication system	High-speed interconnection network
File system	Lustre file system
Operating system	Kylin 2.6.32–431.TH.x86_64

A comprehensive performance study was conducted in our experiments on both real molecular and synthetic datasets. The first real dataset we tested is the Predictive Toxicology dataset (PTE). It is sparse and contains 340 chemical compounds, 24 different atoms, 66 atom types, and 4 types of bonds. We use the type of atoms and bonds as labels. The second real dataset is the AIDS antiviral screen compound dataset from Developmental Therapeutics Program in NCI/NIH. It contains 43,905 chemical compounds. The results of the screening tests can be categorized into three classes: CA: confirmed active; CM: confirmed moderately active; and CI: confirmed inactive. We only use CA class in our tests which consists of 422 molecules (dataset DTP).

The synthetic graph dataset is using a synthetic data generator similar to that described in [12]. A user can set parameters to decide the number of graphs and their average size. We generate three datasets (S1, S2 and S3) for our tests, which consist of 10,000 graphs, 20,000 graphs and 100,000 graphs respectively. More information of these four datasets in shown in Table 2.

**Table 2:** Dataset information

Dataset	Molecules Or Graphs	Average #edges	Largest #edges	Average #vertices	Largest #vertices
PTE	340	28	214	27	214
DTP	422	42	196	40	188
S1	10,000	29	276	26	225
S2	20,000	32	214	30	197
S3	100,000	45	321	38	278

### Parallelization on single node

We try to compare our tool with a wide range of functionally comparable frequent structure miners, such as FSM [12], FFSM [14], gSpan [15] and Gaston [17]. We should note that some of these miners had restrictions regarding the number of labels or were restricted to molecular database. For these algorithm we only publish limited results.

In this part, we have used first three datasets for analysis to show that cmFSM can easily present a better performance than any other famous miners in a relatively low level of parallelization. Table 3 compares results and runtimes among cmFSM and other miners on PTE dataset.

**Table 3:** Results for PTE dataset

MinSup %	2	4	6	8	10	20	30
MinSup Abs	7	14	20	27	34	68	102
Freq graphs	136,949	5935	2326	1323	844	190	68
Runtime in Seconds							
FSM	312.21	11.22	4.12	2.51	1.69	0.66	0.31
FFSM	78.12	5.21	2.01	1.03	0.75	0.58	0.29
gSpan	101.12	7.21	2.31	1.21	0.83	0.65	0.33
Gaston	36.53	2.13	1.01	0.66	0.38	0.12	0.06
cmFSM	97.32	3.28	1.25	0.71	0.49	0.15	0.08
cmFSM_2t	68.23	2.98	1.01	0.63	0.47	0.17	0.09
cmFSM_8t	21.51	1.03	0.42	0.27	0.21	0.08	0.07
cmFSM_32t	6.32	0.44	0.21	0.16	0.14	0.06	0.06

From Table 3, it is not difficult to find that cmFSM presents a significant performance advantage. The last three lines in Table 3 represent that cmFSM starts 2 threads, 8 threads and 32 threads respectively. It can be seen that even the serial version, the runtime of cmFSM is less than gSpan. In addition, as long as we start more than 8 threads, the runtime of cmFSM is less than all other tools. This proves that cmFSM can show better performance than other state-of-the-art miners even if we only hold a few parallel computing resources. Moreover, the consistency of the mining results also demonstrates that our parallel optimization does not affect the correctness of the miners.

**Fig. 6:**
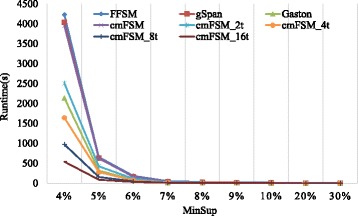
Comparison of some miners for DTP dataset

Fig. 6 reflects the mining conditions on DTP datasets. From Fig. 6, it is not difficult to see that cmFSM can also achieve better performance than any other state-of-the-art miners with a small number of threads on DTP dataset. Also, the mining scale will drastically decrease with the increase in support threshold.

**Fig. 7:**
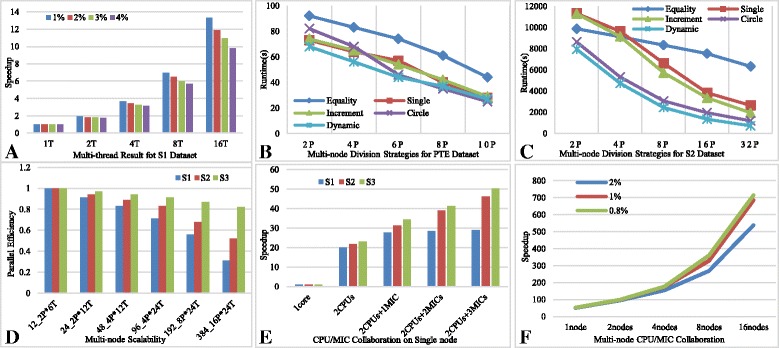
Experiments of parallel optimization. There are six pictures labeled as **a**,**b**,**c**,**d**,**e** and **f** respectively. The “T” in coordinate axis means the number of thread we have started. The “P” in coordinate axis means the number of process we have started. The “1Core” means we have only started one process with single thread to carry out these experiments. The “2CPUs” means we have started one process with 24 threads to carry out these experiments. This is because there are two CPUs with 12 cores on single node of Tianhe-2 supercomputer

The experiments on S1 dataset is shown on Fig. 7a, which reflects the excellent parallel acceleration effect of cmFSM. We have set 1%, 2%, 3% and 4% as support thresholds respectively to form different scale of mining process. Basically, doubling the number of threads, the speedup is doubling too, which is close to the linear speedup. Also, the smaller the support threshold is, the larger the mining scale will be, where cmFSM presents a better parallel efficiency. This means it can be well applied to large-scale mining scenes.

### Multi-node division strategy

In order to compare the advantages and disadvantages of the five division strategies under different computing scenarios, we have experimented on DTP, PTE and S2 datasets, and set 4%, 2% and 1% as support thresholds respectively. Also, in order to eliminate the impact of multi-thread parallelization, we have only started one thread of each process. The Table 4 shows the results on DTP dataset.

**Table 4:** Multi-node results for DTP dataset

Process number	Equality	Single	Increment	Circle	Dynamic
2	3646	3183	3192	3532	3203
3	3584	3174	3185	3493	3213
4	3567	3178	3191	3468	3208
5	3552	3185	3188	3452	3216

From Table 4, it is not difficult to see that the runtime did not be significantly reduced among anyone of these five strategies with the number of process increases. This is because the first single-edge frequent subgraph will pick out 80% of the results on DTP dataset. Thus, the first process will become the bottleneck. Moreover, the equality strategy is undoubtedly the worst strategy. The single and increment strategies are relatively faster, because the first single-edge frequent subgraph will be uniquely allocated to first process. Although the dynamic strategy also has such a division result, it has additional communication scheduling overhead, so that the performance is slightly lower than these two strategies. However, no matter how much the processes are started, the mining time of first single-edge frequent subgraph is always bottleneck on such a special dataset.

Figure 7b and c reflects the multi-node runtimes on PTE and S2 datasets. The condition that more than half of the results are picked out by the first single-edge frequent subgraph will not happen again on these normal datasets, such as PTE and S2.

From Fig. 7b, we can find the equality is also the worst strategy. The single and increment strategies present close performance. The circle strategy gradually shows the advantages of performance with the number of process increases. The dynamic strategy is the best at first, but with the increase in the number of process, its performance begin to slowly weaker than circle. This is due to the fact that the mining process of single-edge frequent graphs can be completed quickly on this dataset, so that as the number of process increases, there are frequent task requests and competitions. Coupled with the communication costs, the dynamic strategy shows weakness compared to circle. Fortunately, this is not an obvious weakness.

From Fig. 7c, we can find that the equality is still the worst strategy on average. At the beginning, single and increment are weaker than equality, which is because a large number of tasks are allocated to the final process when the number of process is small so as to form a bottleneck. Also, it is not difficult to find that the circle is an optimal choice among static strategies when the mining scale is big enough. The single and increment strategies still present close performance and the dynamic strategy is always better than all these static ones. Therefore, it is more recommended to use dynamic strategy, especially in the face of large-scale mining tasks.

### Efficiency of multi-node multi-thread acceleration

In order to evaluate the efficiency of multi-node acceleration, we have experimented on S1, S2 and S3 datasets, and set 1%, 1% and 2% as support thresholds respectively.

The Table 5 shows the result of multi-node scalability. We should note that we have always adopted dynamic strategy in the following experiments because this division strategy can achieve an average best performance among all these five division strategies. By this way, we can simplify following tests.

**Table 5:** Multi-node scalability

	12Core 2P*6 T	24Core 2P*12 T	48Core 4P*12 T	96Core 4P*24 T	192Core 8P*24 T	384Core 16P*24 T
S1	742	408	223	131	83	74
S2	1732	921	487	261	161	104
S3	22,821	11,763	6069	3135	1640	871

Figure 7d indicates the comparison of parallel efficiency on different datasets which we can get from Table 5. we can easily find that the parallel efficiency will be better maintained with the increase in the number of cores for larger datasets, which also means cmFSM can be well applied to large-scale mining scenes.

### CPU/MIC collaboration

We have also used the last three datasets with 1%, 1% and 2% as support thresholds respectively to evaluate the effect of CPU/MIC collaboration on single node. The following Table 6 shows the results. In general, the computing capacity of 2 CPUs should be close to 3 MICs on single node. Therefore, we expect to be 24 times faster with 2 CPUs and achieve 48 times speedup with 2 CPUs and 3 MICs to make full use of computing resources on single node.

**Table 6:** CPU/MIC collaboration on single node

	1Core	2CPUs	2CPUs &1MIC	2CPUs &2MICs	2CPUs &3MICs
S1	7590	382	274	234	208
S2	18,944	875	607	486	410
S3	266,794	11,625	7749	6457	5320

Figure 7e reflects the comparison of speedup on different datasets with different CPU/MIC collaboration modes which we can get from Table 6. We can easily find that the better accelerating effect can be achieved with larger scale mining tasks. Moreover, we obtained more than 50 times speedup finally on S3 dataset, which is better than we expect to get. This is because of a series of optimization means we adopt, such as memory reuse, data transmission optimization and vectorization. In addition, the experiment on S1 dataset quickly reached the bottleneck and there is no obvious difference between 2 MICs mode and 3 MICs mode. This should also be caused by the characteristics of the dataset itself. On this dataset, a large number of tasks are always concentrated in the Host and first MIC coprocessor. However, the accelerating effect of CPU/MIC is still nice in most large-scale mining scenarios.

### Multi-node CPU/MIC collaboration

We have employed the biggest dataset S3 with 2%, 1% and 0.8% as support thresholds respectively to evaluate the effect of CPU/MIC collaboration. The following Table 7 shows the results where each node have made full use of 2 CPUs and 3 MICs.

**Table 7:** Multi-node CPU/MIC collaboration

MinSup	1node	2nodes	4nodes	8nodes
2%	5320	2816	1736	993
1%	18,329	9802	5508	2918
0.8%	43,253	23,026	12,847	6295

Figure 7f reflects the comparison of multi-node speedup on S3 with different support thresholds which we can get from Table 7. We can also easily find that the better multi-node accelerating effect can be achieved with larger scale mining tasks. In overall, all these experiments present weaker and weaker speedup than linear speedup, which is mainly caused by multi-node communication, process competition and synchronization. However, this condition does not affect the excellent scalability of cmFSM under large-scale mining tasks.

## Conclusions

cmFSM is a scalable parallel frequent subgraph mining tool using CPUs and MICs in collaboration. It realizes parallelism of multiple levels and multiple granularities. We first adopt a fine-grained parallel strategy among threads by translating the common recursive mining process into a BFS loop mining process on single node. In addition to some special datasets, cmFSM can obtain near-linear speedup. Second, a coarse-grained parallel strategy is used among processes by dividing single-edge frequent subgraphs. Four kinds of static task dividing (i.e. equality, single, increment, circle) strategies and a supervisor-based dynamic task dividing strategy are implemented to achieve load balancing as much as possible. Some experiments show the dynamic strategy mostly presents better performance than all these static ones, especially in the face of large-scale mining tasks. Also, combining with the multi-thread work on single node, our implementation allows to generate a second level of parallelization by creating multiple threads per MPI process, which shows a promising scalability of massive mining tasks. Third, the collaboration among CPUs and MICs we employ a medium-grained parallel strategy by dividing the double-edge frequent subgraphs which is gotten from one-edge growth of initial single-edge frequent subgraphs. We also back up complex data structures redundantly to avoid the bottlenecks caused by excessive transmission. By memory reuse and making full of the multi-core computing capacity of MIC, we can obtained more than 50 times speedup on single node for some datasets. Also, the multi-node CPU/MIC collaboration presents an excellent scalability under large-scale mining tasks.

Moreover, Experimental evaluation results on several real compound molecular datasets show that cmFSM clearly outperforms the existing state-of-the-art miners even if we only hold a few parallel computing resources, which sufficiently demonstrates the effectiveness of our tool in the field of bioinformatics. However, on some special datasets, which will concentrate most of the mining tasks on a few front single-edge subgraphs, cmFSM will show a great limitation. In this scenario, it will quickly reach the bottleneck, which needs to further our work to solve in the future.

## Abbreviations

FSM: Frequent subgraphs mining
GPU: Graphics Processing Unit
MIC: Many Integrated Core
